# Primary staging and follow-up in melanoma patients – monocenter evaluation of methods, costs and patient survival

**DOI:** 10.1038/sj.bjc.6600428

**Published:** 2002-07-02

**Authors:** U Hofmann, M Szedlak, W Rittgen, E G Jung, D Schadendorf

**Affiliations:** Skin Cancer Unit (German Cancer Research Centre), University Hospital Mannheim, Theodor Kutzer Ufer 1, Mannheim 68167, Germany; Department of Biostatistics (German Cancer Research Centre), Im Neuenheimer Feld 280, Heidelberg 68120, Germany; Department of Dermatology, University Hospital Mannheim, Theodor-Kutzer Ufer 1-3, Mannheim 68167, Germany

**Keywords:** malignant melanoma, follow-up, prognosis, health economy

## Abstract

In a German cohort of 661 melanoma patients the performance, costs and survival benefits of staging methods (history and physical examination; chest X-ray; ultrasonography of the abdomen; high resolution sonography of the peripheral lymph nodes) were assessed at initial staging and during follow-up of stage I/II+III disease. At initial staging, 74% (23 out of 31) of synchronous metastases were first detected by physical examination followed by sonography of the lymph nodes revealing 16% (5 out of 31). Other imaging methods were less efficient (Chest X-ray: one out of 31; sonography of abdomen: two out of 31). Nearly 24% of all 127 first recurrences and 18% of 73 second recurrences developed in patients *not* participating in the follow-up programme. In follow-up patients detection of first or second recurrence were attributed to history and physical examination on a routine visit in 47 and 52% recurrences, respectively, and to routine imaging procedures in 21 and 17% of cases, respectively. Lymph node sonography was the most successful technical staging procedure indicating 13% of first relapses, but comprised 24% of total costs of follow-up in stage I/II. Routine imaging comprised nearly 50% of total costs for follow-up in stage I/II and in stage III. The mode of detecting a relapse (‘patient *vs*. doctor-diagnosed’ or ‘symptomatic *vs* asymptomatic’) did not significantly influence patients overall survival. Taken together, imaging procedures for routine follow-up in stage I/II and stage III melanoma patients were inefficient and not cost-efficient.

*British Journal of Cancer* (2002) **87**, 151–157. doi:10.1038/sj.bjc.6600428 www.bjcancer.com

© 2002 Cancer Research UK

## 

At present, physician's intuition and sense of responsibility, but not solid data, justify the extensive and costly postoperative procedures used to perform follow-up of cutaneous melanoma patients. There is no consensus on the best follow-up program for patients with resected primary melanoma, nor is there much data to help oncologists achieve such agreement (Kersey *et al*, 1985; Conference, 1992; Rumke *et al*, 1992; Orfanos *et al*, 1994; Romero *et al*, 1994; Bassères *et al*, 1995; Shumate *et al*, 1995; Eggermont, 1996; Ross, 1996; Provost *et al*, 1997; Huang *et al*, 1998; Mooney *et al*, 1998; Poo-Hwu *et al*, 1999; Hormbrey *et al*, 2000).

German follow-up guidelines for melanoma call for regular history and physical examination, blood tests and chest X-ray, also for ultrasonography of the abdomen and high resolution ultrasonography of the peripheral lymph nodes (Orfanos *et al*, 1994). However, only few studies deal with the efficacy of sonographic abdominal screening (Bassères *et al*, 1995; Bastien *et al*, 1997), and there are no large scale reports published on the usefulness of ultrasonography of the peripheral lymph nodes for screening purposes. In addition, studies hardly ever analyse the costs of follow-up methods in relation to their screening success, and only few try to show any influence of the mode of recurrence detection on patient survival (Baughan *et al*, 1993; Bassères *et al*, 1995; Shumate *et al*, 1995; Poo-Hwu *et al*, 1999). Based on these facts, there is an urgent need to establish standardised protocols for follow-up that balance costs and use of medical resources with patients' benefits. In the absence of prospective data, this study attempts to pave the way by performing a systematic retrospective evaluation of the records of 661 patients with melanoma stages I–III who had been seen and, for the most part, followed between January 1983 and November 1999 at the Department of Dermatology at the University Hospital Mannheim.

## PATIENTS AND METHODS

### Patient selection

Out of 870 medical records from melanoma patients that could be traced in the archives, 661 records (630 stage I/II, 27 stage IIIA/B, four stage IV patients at time of first diagnosis) from January 1983 to November 1999 were selected as a historical cohort for further evaluation. The year 1983 was chosen as a starting point because existing follow-up guidelines have only been slightly modified since then (addition of lymph node sonography to routine follow-up in 1986; extension of the follow-up period from 8–10 years in 1994). These 661 records met the principal selection criteria which were (a) management of the primary cutaneous melanoma (or of loco-regional recurrence) and (b) proper primary documentation at our clinic. Records were considered for evaluation of initial staging techniques if they contained at least one documented staging result at time of primary excision. For evaluation of follow-up of stage I/II and stage III patients only records that confirmed at least 6 months of follow-up in the outpatients clinic were included. Patients' characteristics which are of known prognostic relevance for localised stage I/II disease (histological types, site of primary melanoma, age and sex) closely resembled those of other historical cohorts from years 1981–1990 (Baughan *et al*, 1993) or 1971–1995 (Mooney *et al*, 1998) (data not shown).

### Initial staging and follow-up

Within 2 weeks of initial physical examination and definite surgical treatment of a primary cutaneous melanoma, patients usually underwent radiodiagnostic imaging procedures including chest X-ray, sonography of the abdomen and high resolution sonography of peripheral lymph nodes. In addition, patients with pT >0.75 mm frequently had scintigraphies of the bones and CT-scans of the brain. Since 1983 the frequency and type of melanoma follow-up procedures at our institution have been adapted to the clinical stage, using the German DDG staging system (Orfanos, 1994) which is close to the UICC 1987 classification system. Routine follow-up for stage I/II patients consisted of physician visits every 3 months during the first 5 years, and every 6 months thereafter until the end of year 8 or recurrence detection. Chest X-ray and sonography of the abdomen were annually done on each patient. Lymph node sonography of peripheral nodes was routinely performed every 6 months during the years 1986–1997 at follow-up of patients in stage I/II. The postsurgical follow-up of patients with loco-regional recurrence were usually extended by increasing the frequency of diagnostic imaging (Chest X-ray+sonography of abdomen twice a year, sonography of lymph nodes four times a year). Until 1997 blood tests (complete blood count, liver transaminases, LDH) were done at every patients' visit during follow-up.

### Data acquisition and analyses

The following information was extracted and entered into a Microsoft ACCESS®97 (SR-21) data base: patients' primary data, survival status, diagnosis of synchronous or metachronous secondary malignant melanoma or other cutaneous malignancies, time points of each physical examination/diagnostic imaging procedure at initial staging and during follow-up of stage I/II or stage patients and their results (negative [neg]; false-positive [fp]; true-positive [tp], time point and detection method of first and/or second relapse). Results and number of all subsequent imaging procedures caused by false-positive results of routine tests during initial staging and follow-up at any clinical stage were carefully collected (repetition of same test, other diagnostic methods, e.g. CT- or NMR-scans). Missing information on mode of recurrence detection or survival status was obtained by means of questionnaires and telephone calls to the patient or surviving close family members. Evaluation was discontinued when patient developed a 2nd recurrence or distant metastatic disease. For descriptive analysis and survival analysis data were transferred from ACCESS® into SPSS® for Windows (Release 10.0.7). This software package also provided algorithms for the Kaplan–Meier method of analysis for actuarial survival, with comparisons between survival curves calculated using the log-rank test. ‘Detection rate’ of a given diagnostic method was defined as the percentage of relapses revealed by this test only and not by any other simpler and cheaper method. The costs of initial staging and follow-up at clinical stage I/II and stage III were calculated on basis of the year 2000 price scale for outpatient treatment (‘GOÄ’) within the German Public Health Care System, neglecting lower price levels during the last 17 years. All subsequent imaging procedures and costs caused by false-positive results obtained from a given diagnostic method were also documented and included in the total cost calculation.

## RESULTS

Based on the entry criteria of this evaluation (see above), data were extracted from 561/661 records. Files of 554 out of 630 stage I/II patients and of 93 stage III patients within a median follow-up time of 4.1 and 1.5 years, respectively, were analysed. The detection mode of recurrent disease could be categorised in 127 stage I/II patients presenting first recurrences, and in all 73 stage III patients developing second recurrences.

### Recurrence pattern

**Figure 1:**
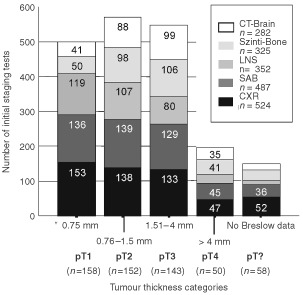
Number of documented initial staging tests performed at the time of primary diagnosis.

In 561 out of 661 patients with primary melanoma an initial staging was performed. Imaging procedures (Figure 1

) detected synchronous metastases in 31 out of 561 patients. Twenty-seven out of 31 patients were upstaged to IIIA/B disease (4.7%), only four patients (0.6%) were shown to be stage IV with asymptomatic distant metastases. After excision of the primary tumour in 630 stage I/II melanoma patients, a total of 127 (20%) FR were identified over time. Distribution of Breslow thickness and recurrence rate among stage I/II patients in our study group correlated well with other study cohorts as shown in Table 1

**Table 1:**
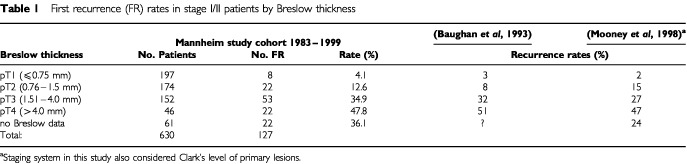
First recurrence (FR) rates in stage I/II patients by Breslow thickness

**Figure 2:**
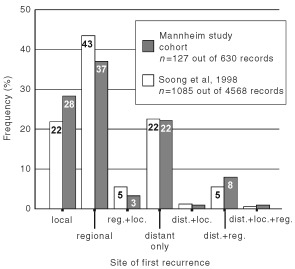
Distribution pattern of 127 first recurrences in 630 FU I/II patients. The recurrence pattern was classified into *local* (satellites or in-transit metastases), regional (regional lymph nodes), distant (viscera; distant (sub-)cutis or lymph nodes) or combinations of these locations.

. Five hundred and fifty-four out of 630 patients participated in the follow-up programme for more than 6 months. Ninety-five out of 127 first relapses were detected in the follow-up of patients in stage I/II with 88 recurrences (90.7%) being noted in the first 5 years and with 75 (77.3%) of the metastases detected by the end of third year. Eight out of 197 (4.1%) patients with a ‘low risk’ melanoma (pT ⩽0.75 mm) relapsed; only two after the fifth year. The metastatic pattern of first relapse is shown in Figure 2

and was similar to other reports (Kersey *et al*, 1985; Fusi *et al*, 1993; Soong *et al*, 1998). Ninety-three patients with surgically resected loco-regional metastases (24 initial stage IIIA/B and 69 former stage I/II patients) were enrolled into the follow-up programme, in 60 out of 93 stage III patients a relapse was documented (64.5%) within a median time of 7.8 months.

### Efficacy of staging methods

The detection rate including the number of false-positive results of each staging method separated by the clinical phase (initial staging, follow-up stage I/II and III) is given in Table 2

**Table 2:**
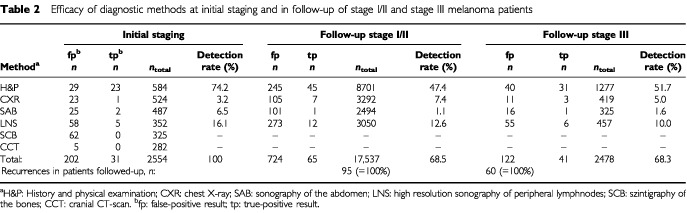
Efficacy of diagnostic methods at initial staging and in follow-up of stage I/II and stage III melanoma patients

. At initial staging, 2554 imaging procedures were performed in 561 patients yielding 31 metastases (true-positive) and 202 false-positive results leading to further technical examinations. In follow-up of stage I/II patients 30 metastases (32%) were detected by the patient and triggering a premature visit, however, 45 of the remaining 65 metastases detected at this stage were detected by the doctor (Table 2). At any phase of melanoma staging and follow-up patient' history and physical examination was the most successful diagnostic tool indicating the vast majority of all relapses (around 70%) in patients attending the follow-up program. Although lymph node sonography was the best performing method among the imaging procedures, detection rate was substantially lower (between 15–20%) compared to physical examination and detection rate of lymph node sonography notably decreased at the different phases of melanoma disease. Chest X-ray and sonography of the abdomen showed extremely low detection rates (below 10%) when used for routine follow-up in stage I/II and stage III patients (Table 2). Overall, the detection of 65 out of 95 first (68.5%) and 41 out of 60 second relapses (68.3%) could be attributed to the scheduled follow-up activities. Thirty out of 127 first relapses (24%) developed in patients not enrolled into the follow-up programme at the time of diagnosis. At initial staging, 325 scintigraphies of the bones and 282 cranial CT scans were performed without revealing any metastases whereas lymph node sonography was remarkably effective (16% detection rate (five out 31 metastases detected)) (Table 2).

### Cost-efficiency of staging methods

Absolute and relative expenses (including costs caused by false-positive results) of each staging method were summarised (Table 3

**Table 3:**
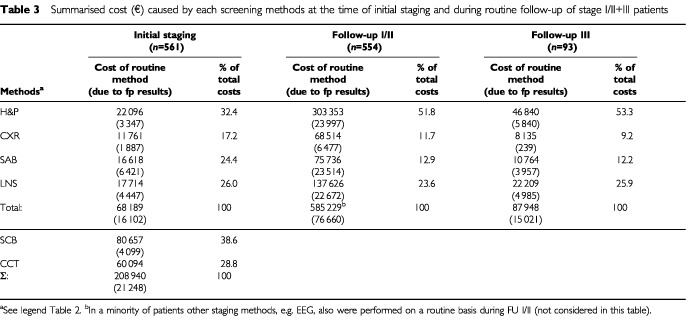
Summarised cost (€) caused by each screening methods at the time of initial staging and during routine follow-up of stage I/II+III patients

). Absolute costs for clinical assessment were the highest in all clinical phases making up to 53% of total costs for follow up. Detection rates were high (Table 2). Costs for physical assessment ranged between €1100 (at initial staging) to €7300 (follow-up in stage I/II) per detected metastasis. Sonography of the lymph nodes proved to be the most cost consuming technical screening method with about 25% of total expenses at each phase of follow-up. Costs ranged between €4400 at initial staging to €13 300 (follow-up of stage I/II) per detected metastasis. In contrast, total costs for screening by chest X-ray ranged between 17.2 to 9.2% (Table 3), however, detection rates were low (Table 2) and costs to detect a metastasis ranged between €2800 (in stage III) to €13 500 at initial staging. Among initial staging methods, scintigraphy of bones and cranial CT-scans were most expensive and comprised 67.4% of total costs. Considering ‘detection rate’ and ‘relative costs’, the efficiency ratio for each method clearly demonstrated the physical examination to be superior to all other staging at all follow-up phases. The costs for each relapse detected within each risk category of primary tumour during follow-up in stage I/II (Table 4

**Table 4:**
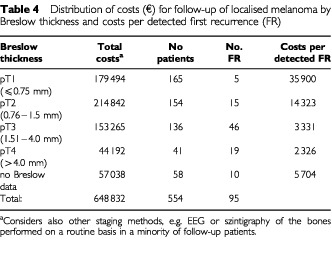
Distribution of costs (€) for follow-up of localised melanoma by Breslow thickness and costs per detected first recurrence (FR)

) varied from €35 900. (pT⩽0.75 mm) to €2326. (pT>4.0 mm). Due to the low rate of relapses in patients enrolled into follow-up program in stage I/II in 5th and following years, the mean price per detected recurrence drastically increased from €5806 (±€1.289, s.d.; years 1–4) to €18 558 (±€6.706, s.d.; years 5–8).

### Survival analyses

**Figure 3:**
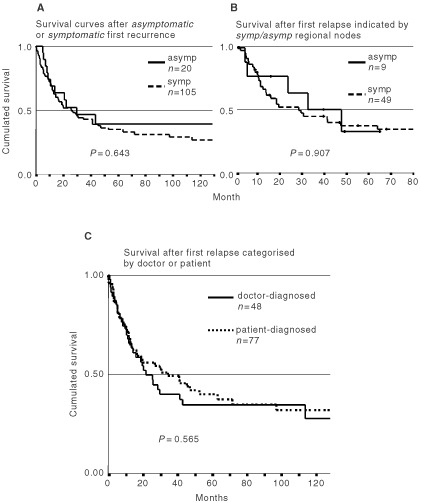
Kaplan–Meier curves after detection of symptomatic (symp) and asymptomatic (asymp) first recurrences in stage I/II patients (**A**) Comparison of survival curves between first relapse patients which were grouped by whether the first recurrence was detected due to routine imaging methods in an asymptomatic stage or clinically by patients/physicians due to symptoms (*P*=0.643, log-rank-test). (**B**) Sonography of the regional lymph node indicated a first relapse in 9 nine out of 95 stage I/II patients attending follow-up. Their survival was compared to *n*=49 patients with symptomatic regional nodes (*P*=0.907, log-rank-test). (**C**) 125 patients were grouped according to the mode of detection of FR (‘doctor-diagnosed’ *vs* ‘patient-diagnosed’; *P*=0.565, log-rank-test).

Survival after relapse was analysed by mode of detection of relapse. There was no difference in survival between patients with symptomatic relapse (84%) and asymptomatic relapse (16%; Figure 3a

). Also the small subset of patients during follow-up in stage I/II in whom a first recurrence was detected by lymph node sonography (*n*=9) did not survive significantly longer than patients suffering from symptomatic nodes (Figure 3b). Comparison of survival times of patients with first relapse classified into ‘doctor-diagnosed’ (*n*=48) or ‘patient-diagnosed’ (*n*=77, including symptomatic ‘no follow-up’ patients) did not show significant survival advantages for any group (Figure 3C). Survival analyses were performed in patients attending follow-up in stage III in the same way (‘asymptomatic’ *vs* ‘symptomatic’; ‘doctor-’ *vs* ‘patient-diagnosed’) and did not show any survival advantages (data not shown).

## DISCUSSION

### Initial staging

Among the radiologic diagnostic methods employed at the various stages of melanoma disease, the examinations with chest X-ray and sonography of the abdomen and also, frequently, the bone scans and cranial CT scans performed for initial staging in medium/high risk primary tumours, have received much criticism because their diagnostic profit is low and they yield a high rate of false-positive results (Kersey *et al*, 1985; Ardizzoni *et al*, 1987; Zartman *et al*, 1987; Khansur *et al*, 1989; Terhune *et al*, 1998). Our data at initial staging argue for the omission of both, chest X-ray and sonography of abdomen at initial staging since the efficiency ratios of these two methods were far below 0.5 – reflecting a very disadvantageous ratio between performance and costs of this staging method. Cranial CT and bone scans obviously have no place in the routine initial staging since they are quite expensive (about 67% of total costs of the initial staging) and detected no recurrence at all in 282 and 325 patients, respectively. Only lymph node sonography had some diagnostic value revealing lymph node metastases that had not been detected by the physical examination previously. Kersey *et al* (1985) reported similar results with two true-positive pedal lymphangiograms in 73 patients with a lower limb melanoma out of more than 300 patients initially staged. A detection rate of 16% (five out of 31 initial stage III/IV patients) argues for lymph node sonography to be included in the routine work-up at initial staging. However, with the increasing use of sentinel node biopsy technique in primary ‘risk melanomas’ at time of primary melanoma excision (Reintgen *et al*, 1998), lymph node sonography may turn out to be redundant.

### Follow-up stage I/II

In 24% of all 127 patients with first relapse the symptoms appeared either in patients who had never participated in the follow-up programme or had prematurely discontinued or had completed the whole course of follow-up examinations. This seems to be a surprisingly high number and has never been reported in any previous evaluation (Bassères *et al*, 1995; Bastien *et al*, 1997; Mooney *et al*, 1998; Poo-Hwu *et al*, 1999). These data suggest that thorough and reliable surveillance of melanoma patients is, in reality, hardly possible because of the often erratic and unpredictable course of the disease and because of the difficulty of attaining full patient compliance.

In patients attending the FU I/II program, about 68% of first relapses were detected due to scheduled follow-up activity. This is a significantly higher yield than reported by Dicker *et al* (1999) who found only 26% of first recurrences detected at the follow-up clinic. Baughan *et al* (1993), Kersey *et al* (1985) as well as Bassères *et al* (1995) found 41, 44 and 50%, respectively, of all first relapses to be detected by routine follow-up activity considering only the purely doctor-diagnosed recurrences. Routine imaging procedures contributed 21% to the overall detection rate at scheduled visits in this study. This detection rate is mainly due to the routine use of lymph node sonography which was the imaging method with the highest yield of all technical staging methods (12 out of 95 relapses). In contrast, the yield of routine technical examinations in stage I/II patients have previously been reported to be much lower between 4–11%, with studies mostly reporting the use of chest X-ray and sonography of the abdomen, but none using sonography of the lymph nodes (Kersey *et al*, 1985; Goerz *et al*, 1986; Bassères *et al*, 1995; Weiss *et al*, 1995; Bastien *et al*, 1997; Mooney *et al*, 1998).

### Follow-up stage III

This is the first study separately analysing the efficacy of routine imaging procedures in stage III patients detecting a second relapse. Only a recent retrospective study reported on the detection mode of SR (‘patient’ *vs* ‘doctor’-diagnosed) in FU III patients (Poo-Hwu *et al*, 1999), however, the performance of routine imaging procedures was not discussed. Although the frequency of imaging procedures being employed in follow-up of stage III patients was doubled compared to stage I/II according to the German follow-up recommendations, this did not result in an increased detection rate. In fact the efficiency ratio decreased whereas routine physical examinations on a regular visit became the clearly dominating mode of recurrence detection in stage III patients (31 out of 60 second relapses; Table 2). These observations may be related to the metastatic pattern in our 73 stage III patients diagnosed at the time of second relapse: Although 42% of all patients with a relapse had distant organs involved, just 19% of the patients had a distant relapse only, and in 81% of all second relapses at least on loco-regional site was again involved which was easily be detected by physical examination at the scheduled visit.

### Cost efficiency/patients survival

Previous papers have estimated the costs of treating cutaneous melanoma, with more (Bassères *et al*, 1995; Mooney *et al*, 1997) or less (Fader *et al*, 1998; Tsao *et al*, 1998) emphasis on the costs of melanoma follow-up. To evaluate a particular screening method from a health economic point of view, our study focused on the relationship of efficacy to detect metastases during initial staging and follow-up in stage I/II and stage III in comparison to the overall costs and the cost per metastasis detected for each screening method. Although financial resources allocated to physical examinations proofed to be the absolutely highest (Table 3), the costs per detected metastasis were in all phases the lowest. This finding is in agreement with a report by French authors (Bassères *et al*, 1995).

Sonography of the lymph nodes was the most efficient technical method to detect relapses (range: 10–16%) in all phases of follow-up. This is in line with recent reports suggesting that lymph nodes sonography improves early detection of locoregional metastases (Tregnaghi *et al*, 1997; Blum *et al*, 2000). The efficiency-costs ratio in our study was best at initial staging and the follow-up in stage III.

In conclusion, the results of this retrospective study cast serious doubt on the efficiency of expensive routine imaging procedures at initial staging and during early phases of melanoma disease. Technical screening methods detected 20 occult recurrences out of 95 first relapses in the entire stage I/II cohort, but comprised roughly 50% of follow-up costs in this monocentric German setting. Since survival seems not to be affected by follow-up activities at present, there is little justification for such an investment. Prospective evaluation of intensive follow-up programs in breast carcinoma has similarly shown a lack of effect on health related quality of life and survival (GIVIO-Investigators, 1994; Rosselli Del Turco *et al*, 1994), and data on effectiveness of follow-up in colo-rectal cancer gave inconsistent results (Kjeldsen *et al*, 1997; Rosen *et al*, 1998; Northover, 2000), thus indicating that prospective studies would shed more light on the real value of various follow-up procedures for melanoma patients.

Follow-up of melanoma patients with ‘low-risk’ melanomas (<0.75 mm) is highly ineffective and therefore cost-intensive (Table 4). With increasing tumour thickness costs per detected metastasis dropped. There is no question about the importance of physical examinations for patients' education, reassurance, detection of surgically treatable relapses and other primary melanomas. Based on the results of this retrospective study a recommended schedule for initial screening and follow-up is given in Table 5

**Table 5:**
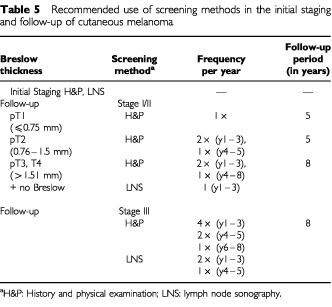
Recommended use of screening methods in the initial staging and follow-up of cutaneous melanoma

.
